# Extraction, Identification, and Preliminary Investigation of the Antihypertensive Mechanism of ACE-Inhibitory Peptides from *Apocynum venetum* L.

**DOI:** 10.3390/foods15132396

**Published:** 2026-07-06

**Authors:** Huiling Huang, Zhichao Yang, Lin Ye, Xujie Hou, Yiming Jia, Shenghuizi Chen, Ying Huang

**Affiliations:** Key Laboratory of Deep Processing of Characteristic Agricultural Products in Southern Xinjiang, College of Food Science and Engineering, Tarim University, Alar 843300, China

**Keywords:** *Apocynum venetum*, ACE-inhibitory peptides, structural characterization, LC–MS/MS, molecular docking, antihypertensive mechanism

## Abstract

In this study, *Apocynum venetum* was employed as the raw material to optimize protein extraction and enzymatic hydrolysis processes for the preparation of highly active angiotensin-converting enzyme (ACE)-inhibitory peptides, achieving an ACE inhibition rate of 92.34%. Multispectral analyses and microstructural characterization demonstrated that enzymatic hydrolysis induced the unfolding of protein secondary structures, resulting in a looser and more porous morphology enriched with characteristic amino acids. A total of 2567 peptide sequences were identified by LC–MS/MS, among which 18 potential bioactive peptides were screened. Molecular docking analysis revealed that these peptides interact with the active site of ACE primarily through hydrogen bonding and hydrophobic interactions, with WLRDFL exhibiting the strongest binding affinity. This study systematically elucidates the structural characteristics and antihypertensive molecular mechanisms of ACE-inhibitory peptides derived from *Apocynum venetum*, providing both theoretical insights and experimental support for the development of natural antihypertensive functional foods and the high-value utilization of this plant.

## 1. Introduction

Hypertension, a highly prevalent chronic cardiovascular disorder worldwide, has become a major public health challenge threatening global health. According to statistics released by the World Health Organization (WHO) in 2023, Hypertension affects over 15 billion people worldwide, with nearly half of these cases remaining inadequately controlled [1]. The combination of high prevalence and low control rates imposes a substantial burden on healthcare systems globally. Data from the National Center for Cardiovascular Diseases indicate that the prevalence of hypertension in China has risen to 31.6%, affecting approximately 270 million people, The number of hypertensive patients in China accounts for approximately one-quarter of the global total [2]. Notably, the disease is increasingly characterized by earlier onset and a more insidious progression. Among young and middle-aged populations, risk factors such as high-sodium diets, physical inactivity, obesity, and excessive psychological stress have contributed to a continuous increase in incidence.

Conventional antihypertensive therapies primarily include six major classes: angiotensin-converting enzyme inhibitors (ACEIs), angiotensin II receptor blockers (ARBs), diuretics, α-adrenergic antagonists, β-adrenergic blockers, and calcium channel blockers (CCBs) [3]. Angiotensin-converting enzyme (ACE), a zinc-dependent peptidase, plays a pivotal role in blood pressure regulation [4]. ACE is a membrane-bound protein widely distributed in vascular endothelial and epithelial cells of mammalian tissues, including the heart, lungs, kidneys, brain, and small intestine, and is also present in body fluids such as plasma [5].

Compared with chemically synthesized antihypertensive drugs, food-derived ACE-inhibitory peptides exhibit superior safety profiles and fewer adverse effects. In contrast, natural, food-derived ACE-inhibiting peptides, which originate from the hydrolysis of plant proteins, exhibit excellent biocompatibility and extremely low toxicity. They exert a mild antihypertensive effect, lowering only abnormally elevated blood pressure levels without causing hypotension in healthy individuals, making them an ideal bioactive substance for dietary intervention in individuals with prehypertension. Studies have demonstrated that ACE-inhibitory peptides possess potent inhibitory activity against ACE, exert significant antihypertensive effects, and do not influence normal blood pressure, indicating minimal side effects and promising application potential [6,7]. Previous research has identified ACE-inhibitory peptides with antihypertensive activity from diverse food sources, including dairy proteins, marine organisms, meat products, and plant proteins [8]. Therefore, the development of natural, safe, and effective food-derived ACE-inhibitory peptides represents an emerging and promising strategy for antihypertensive therapy.

*Apocynum venetum*, a perennial herbaceous plant belonging to the family Apocynaceae, exhibits remarkable adaptability to saline–alkaline environments [9]. It is widely distributed across arid and semi-arid saline regions of northwestern, northern, and northeastern China, including Xinjiang, Inner Mongolia, Gansu, and Qinghai, and is also found in Central Asian regions such as Russia and Mongolia. Owing to its abundant reserves and strong regenerative capacity, *Apocynum venetum* represents a characteristic medicinal and edible plant resource in China, as well as an important ecological and economic crop for saline–alkaline lands, providing a reliable raw material basis for the development of natural bioactive compounds [10].

*Apocynum venetum* has a long history of medicinal use. In traditional Chinese medicine, it is described as having a cool nature and a bitter-sweet taste, primarily acting on the liver and heart meridians, with functions including calming the liver, tranquilizing the mind, clearing heat, and promoting diuresis [11]. It is commonly used to alleviate symptoms such as dizziness and vertigo caused by hyperactivity of liver yang, palpitations, insomnia, edema, and dysuria. As a traditional medicinal herb, *Apocynum venetum* exhibits multiple physiological activities, including antihypertensive, hypolipidemic, and antioxidant effects. A well-known folk adage states, “Hypertension is not to be feared; one jin of *Apocynum venetum* over three years suffices,” reflecting its longstanding ethnopharmacological significance [12].

Modern pharmacological studies [13,14,15] have confirmed that *Apocynum venetum* exerts multi-target and multifunctional bioactivities. Its flavonoids and polyphenols can scavenge reactive oxygen species and inhibit lipid peroxidation, thereby alleviating oxidative damage to vascular endothelium. Polysaccharides and alkaloids have been shown to suppress the release of inflammatory mediators, mitigate chronic inflammation in vascular walls, and improve vasodilatory function. In addition, proteins and peptides derived from *Apocynum venetum* have demonstrated antihypertensive properties; preliminary work by our research group revealed that enzymatic hydrolysates of *Apocynum venetum* proteins significantly inhibit ACE activity.

At present, studies on plant-derived ACE-inhibitory peptides have mainly focused on common crops such as soybean, oat, and goji berry [16], whereas research on the targeted preparation, structural characterization, and mechanistic elucidation of ACE-inhibitory peptides from *Apocynum venetum* proteins remains scarce. Its potential as a source of antihypertensive bioactive peptides has yet to be systematically explored. In recent years, the development of natural antihypertensive compounds centered on ACE-inhibiting peptides has become a hot topic in the field of functional foods. Compared with chemically synthesized antihypertensive drugs, which are prone to causing side effects such as dry cough and angioedema, plant-derived ACE-inhibiting peptides offer distinct advantages, including low toxicity, good biocompatibility, and multiple physiological activities such as antioxidant properties, making them ideal natural candidate molecules to replace traditional antihypertensive agents. Currently, extensive research both domestically and internationally has focused on the targeted preparation and elucidation of mechanisms of action in plants with dual medicinal and food uses: through in vitro enzymatic hydrolysis screening of maca proteins, a mixed-type inhibitory peptide, RSRGVFF (IC_50_ = 5.01 μM), was obtained, whose inhibitory effect depends on the interaction between the N-terminal arginine and the zinc-binding motif of ACE [17]. Enzymatic hydrolysis of moringa leaf composite protein yielded competitive inhibitory short peptides such as ILVDR and IPPAYSK, among which the low-molecular-weight fraction (<3 kDa) exhibited the highest activity [18]. Machine learning was used to optimize the enzymatic hydrolysis process of fermented black sesame, resulting in the identification of ITAPHW, which possesses dual antioxidant and ACE-inhibitory activities [19]. Hydrolysis of walnut protein with chymotrypsin yielded the tripeptide YHP, whose hydrophobic amino acid backbone stabilizes the ACE complex [20]. Research on red date protein has remained limited to the in vitro simulated digestion screening stage, lacking a validation system for active peptides in the animal gut; its oral stability and in vivo mechanism of action remain unclear [21]. The aforementioned studies cover a wide range of plant resources, including nuts, oilseeds, medicinal and edible roots and rhizomes, and jujubes, establishing a standardized research paradigm of “enzymatic preparation—separation and purification—virtual screening—molecular docking.” However, existing raw materials are predominantly concentrated on major cash crops, and there remains a significant gap in the discovery of bioactive peptides from distinctive wild medicinal plants.

A synthesis of recent reviews and specialized studies reveals three core limitations in the current development of plant-derived ACE-inhibitory peptides: First, most studies rely solely on static in vitro hydrolysis models, neglecting the actual physiological conditions in vivo—such as degradation by gastrointestinal enzymes and intestinal absorption following oral administration—resulting in a significant decline in the activity of high-potency peptides identified in vitro once they enter the body. Second, existing raw material screening suffers from severe homogenization, with very little attention paid to wild medicinal plants—such as *Apocynum venetum*—that possess both a history of traditional medicinal use and abundant protein resources but have not yet been systematically developed. The potential ACE-inhibitory peptide sequences, mechanisms of action, and digestive stability in *Apocynum venetum* have not been reported. Third, most studies have focused solely on analyzing in vitro inhibition kinetics, lacking a multidimensional, integrated mechanism validation system that incorporates 3D-QSAR quantitative structure-activity relationships, molecular dynamics simulations, and isothermal titration calorimetry. Compared to previously reported studies on plant-derived ACE-inhibitory peptides from sources such as maca, moringa, black sesame, walnuts, and red dates, this study utilized protein from the distinctive wild medicinal plant *Apocynum venetum* as the raw material and optimized protein extraction and composite enzymatic hydrolysis processes to prepare highly active ACE-inhibitory peptides. By conducting multidimensional structural characterization of the peptides, determining their amino acid sequences, and utilizing molecular docking to elucidate the binding characteristics of the active peptides with ACE and their antihypertensive molecular mechanisms, this study fills the gap in systematic research on ACE-inhibiting peptides derived from *Apocynum venetum*. It also provides a comprehensive set of process and theoretical foundations for the development of functional proteins from niche medicinal plants and the creation of natural antihypertensive factors.

## 2. Materials and Methods

### 2.1. Materials and Instruments

#### 2.1.1. Materials

*Apocynum venetum* samples were collected in August 2025 from the forest farm of the Tenth Regiment, First Division of the Xinjiang Production and Construction Corps in Alar City, Xinjiang, China. After collection, the samples were naturally air-dried, impurities were removed, and the purified leaves were retained for subsequent experiments.

Ethyl acetate (Cat. No. Z21-4) was purchased from Tianjin Yongsheng Fine Chemical Co., Ltd. (Tianjin, China) Hydrochloric acid (Cat. No. H65741), sodium hydroxide (Cat. No. k7-1), sodium dihydrogen phosphate (Cat. No. J1-6), ethanol (Cat. No. Z23-1) and n-hexane (Cat. No. Z23-3) were obtained from Tianjin Xinbote Chemical Co., Ltd., (Tianjin, China), Tianjin Beilian Fine Chemicals Development Co., Ltd., (Tianjin, China), Tianjin Zhiyuan Chemical Reagent Co., Ltd., (Tianjin, China), and Fuchen (Tianjin) Chemical Reagent Co., Ltd., (Tianjin, China), respectively. Acetonitrile (Cat. No. Z19-9) was supplied by Tianjin Aopusheng Chemical Co., Ltd. (Tianjin, China) Pepsin (Cat. No. S36152), trifluoroacetic acid (TFA, Cat. No. W12569) and sodium acetate (Cat. No. V33677) were purchased from Shanghai Yuanye Biotechnology Co., Ltd. (Shanghai, China) Methanol (Cat. No. M116115) was obtained from Shanghai Aladdin Biochemical Technology Co., Ltd. (Shanghai, China) Potassium persulfate (Cat. No. P823296) and acetic acid (Cat. No. A201895) were provided by Shanghai Macklin Biochemical Co., Ltd. (Shanghai, China) Trypsin (Cat. No. T8150), acidic protease (Cat. No. B8411), alkaline protease (Cat. No. B8361), hippuryl–histidyl–leucine (HHL, Cat. No. IH1580), acetone (Cat. No. IS4120), DPPH (Cat. No. ID1240) and ABTS (Cat. No. SA5340) were purchased from Beijing Solarbio Science & Technology Co., Ltd. (Beijing, China). A mixed standard of 17 amino acids (Cat. No. CDAA-M-270051-SM-1ML) was obtained from Shanghai Anpu Experimental Technology Service Co., Ltd. (Shanghai, China) Triethylamine (Cat. No. 767447) and phenyl isothiocyanate (Cat. No. 715983) were supplied by Saen Chemical Technology (Shanghai) Co., Ltd. (Shanghai, China) Potassium bromide (Cat. No. p816700) was purchased from Shandong Keyuan Biochemical Co., Ltd. (Laizhou, China) Angiotensin-converting enzyme (ACE, Cat. No. SAE0075 0.5UN) was obtained from Sigma-Aldrich (Merck, Germany). LC-MS-grade formic acid (Cat. No. 28905), acetonitrile (Cat. No. 047138.m1) and trifluoroacetic acid (Cat. No. 031771.22) were purchased from Thermo Fisher Scientific (Waltham, MA, USA).

#### 2.1.2. Instruments

A UV–visible spectrophotometer (Model: UV-2450) was purchased from Shimadzu Corporation, Kyoto, Japan. A high-performance liquid chromatograph (Model: LC-20AT) was supplied by Shimadzu Corporation, Kyoto, Japan. A multi-mode microplate reader (Model: Synergy H1) was obtained from BioTek Instruments, Inc., Winooski, VT, USA. A Fourier transform infrared spectrometer (Model: Spectrum Two^TM^ FT-IR) was purchased from PerkinElmer Inc., Waltham, MA, USA.

A field emission scanning electron microscope (Model: FEI Apreo S), fluorescence spectrophotometer (Model: Lumina, Illumina, San Diego, CA, USA), ultra-high-performance liquid chromatograph (Model: Thermo Scientific™ Vanquish™ Neo UHPLC), and Orbitrap mass spectrometer (Model: Thermo Scientific Orbitrap Astra) were all supplied by Thermo Fisher Scientific, Waltham, MA, USA.

A high-speed refrigerated centrifuge (Model: TGL-20BR) was obtained from Shanghai Anting Scientific Instrument Factory, Shanghai, China. An electronic analytical balance (Model: LE-2002E) was purchased from Mettler-Toledo Instruments (Shanghai) Co., Ltd., Shanghai, China. A digital constant-temperature magnetic stirring water bath (Model: HH-6A) was supplied by Changzhou Ronghua Instrument Manufacturing Co., Ltd., Changzhou, Jiangsu, China. A vacuum freeze dryer (Model: Lab-1C-80) was obtained from Boyikang (Beijing) Instrument Co., Ltd., Beijing, China.

### 2.2. Technical Route

**Figure 1:**
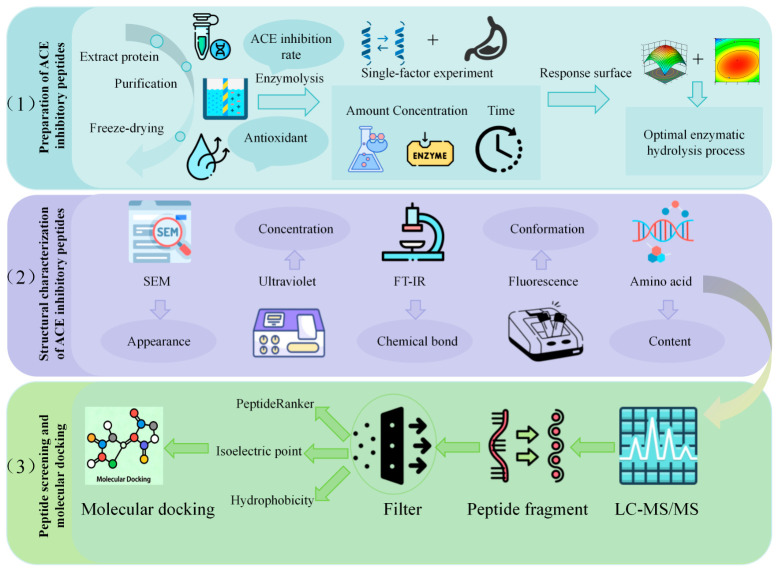
Experimental Workflow. Experimental workflow for obtaining and characterizing ACE-inhibitory peptides from *Apocynum venetum* (**1**) Extraction, purification, and enzymatic hydrolysis of proteins, with process optimization via single-factor and response surface experiments. (**2**) Structural characterization of peptides using SEM, UV, FT-IR, fluorescence, and amino acid analysis. (**3**) LC-MS/MS identification of peptide fragments, followed by multi-parameter filtering and molecular docking to screen for potent ACE inhibitors.

The technical roadmap of this study is illustrated in Figure 1.

### 2.3. Extraction of ACE-Inhibitory Peptides from Apocynum venetum and Optimization of Enzymatic Hydrolysis Conditions

#### 2.3.1. Extraction of Apocynum venetum Proteins

**Figure 2:**
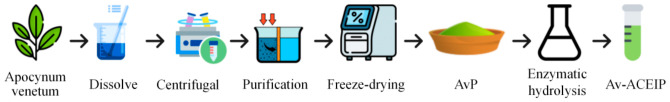
Protein Extraction Process of *Apocynum venetum*.

The extraction of *Apocynum venetum* protein was carried out based on the method reported by Ashok N et al. [22], with slight modifications. Briefly, dried *Apocynum venetum* samples were pulverized and passed through an 80-mesh sieve. It was defatted using n-hexane, followed by drying for subsequent use. The powder was then mixed with 0.1 mol/L NaOH solution at a solid-to-liquid ratio of 1:20 (g:mL) and subjected to ultrasonic water bath extraction at 45 °C for 4 h. The extract was centrifuged at 6000 r/min for 20 min, and the supernatant was collected. The pH of the supernatant was adjusted to 3.5 using 1 mol/L HCl and allowed to stand for 1 h, followed by centrifugation at 6000 r/min for 20 min to collect the precipitate. The precipitate was then re-dissolved and adjusted to pH 7.0 using 0.1 mol/L NaOH. Subsequently, 75% acetone was added for decolorization, and the mixture was maintained for 12 h. After centrifugation at 6000 r/min for 10 min, the precipitate was collected and washed twice with deionized water to remove residual acetone. The resulting sample was transferred into a dialysis bag and dialyzed against deionized water for 24 h, with the water replaced 3–5 times during the process. Finally, the dialyzed protein solution was freeze-dried for 48 h to obtain purified *Apocynum venetum* protein. The technology roadmap is shown in Figure 2.

#### 2.3.2. Preparation of ACE-Inhibitory Peptides from Apocynum venetum by Enzymatic Hydrolysis

The preparation of ACE-inhibitory peptides from *Apocynum venetum* protein was conducted with reference to the method of Lu X [23], with slight modifications. Briefly, the crude protein extract was mixed with ultrapure water at a solid-to-liquid ratio of 1:100 (*w*/*v*) and thoroughly homogenized to obtain a protein solution. Four different proteases were separately added, and enzymatic hydrolysis was performed under their respective optimal temperature and pH conditions, with an enzyme dosage of 5000 U/g for 1 h. The reaction temperatures of different proteases are shown in Table 1.

Upon completion of hydrolysis, the reaction was terminated by heating in a boiling water bath for 15 min to inactivate the proteases. The mixture was then cooled, and the pH was adjusted to neutral. After centrifugation, the supernatant was collected as the crude extract of *Apocynum venetum* ACE-inhibitory peptides. The obtained hydrolysate was subsequently freeze-dried and stored at −80 °C for further use.

#### 2.3.3. Determination of ACE Inhibitory Activity

The inhibitory activity of ACE inhibitors was determined using a UV–visible spectrophotometer, following the method described by Ko J [24]. Specific methods are shown in Table 2. The ACE inhibitory rate was evaluated using the hippuryl–histidyl–leucine (HHL) assay. In this method, ACE catalyzes the hydrolysis of HHL to produce hippuric acid and histidyl–leucine. Hippuric acid exhibits a characteristic absorption peak at a specific wavelength, and the ACE inhibitory activity was calculated based on the change in absorbance.

Post-Treatment Procedure:

Extraction: Transfer 1 mL of the upper ethyl acetate phase and evaporate to dryness in a boiling water bath at 100 °C.

Dissolution: Add 1 mL of deionized water and mix thoroughly by vortexing.

Measurement: Determine the absorbance at 228 nm.

The ACE inhibitory activity was calculated as follows:

(1) Inhibition rate (%) = [(B − A)/(B − C)]×100%

where *A* represents the absorbance of the sample containing ACE-inhibitory peptide extract, HHL, and ACE solution; *B* represents the absorbance of the control containing HHL and ACE solution but without the peptide extract; and *C* represents the absorbance of the blank containing only the peptide extract and HHL without ACE.

#### 2.3.4. Single-Factor Experimental Design

Single-factor experiments were conducted by independently varying substrate concentration (1%, 2%, 3%, 4%, and 5%), enzyme dosage (4000, 4500, 5000, 5500, and 6000 U), and hydrolysis time (40, 50, 60, 70, and 80 min), while keeping other conditions constant. Each level was tested in triplicate.

The ACE inhibitory activity of each experimental group was determined, and the influence trends of individual factors on ACE inhibition were analyzed to preliminarily identify the optimal range of process parameters. All experimental data were statistically analyzed using SPSS software, and differences were considered statistically significant at *p* < 0.05.

#### 2.3.5. Experimental Design and Process Optimization Using Response Surface Methodology

Based on the results of the single-factor experiments, substrate concentration, enzyme dosage, and hydrolysis time were selected as independent variables, while ACE inhibitory rate was defined as the response value. A Box–Behnken design (BBD) was employed to construct the response surface experiment.

Regression analysis was performed using Design-Expert 13 software to establish a quadratic regression model and to evaluate the effects of individual factors and their interactions on ACE inhibitory activity. Response surface plots and contour maps were generated to determine the optimal enzymatic hydrolysis conditions.

Finally, validation experiments were carried out in triplicate to verify the reliability and stability of the optimized process parameters.

### 2.4. Structural Characterization of ACE-Inhibitory Peptides from Apocynum venetum

#### 2.4.1. Endogenous Fluorescence Spectroscopy Analysis

The intrinsic fluorescence spectra were measured with reference to the method of Ashraf J [25], with slight modifications. *Apocynum venetum* protein and ACE-inhibitory peptide samples were dissolved in phosphate-buffered saline (PBS) to prepare a solution with a concentration of 0.2 mg/mL.

The excitation wavelength was set at 295 nm, and the emission spectra were recorded over the range of 300–400 nm. The scanning speed was 240 nm/min, and the slit width was 2.5 nm. PBS was used as the blank control. The fluorescence emission spectra were recorded, and changes in fluorescence intensity as a function of wavelength were analyzed to characterize the intrinsic fluorescence properties of the samples.

#### 2.4.2. Fourier Transform Infrared (FT-IR) Spectroscopy Analysis

FT-IR analysis was performed with reference to the method of Zolqadri R [26], with minor modifications. The sample was thoroughly mixed and ground with dried potassium bromide (KBr) at a mass ratio of 1:100, followed by compression into pellets.

The prepared pellets were analyzed using an infrared spectrometer under the following conditions: scanning range of 4000–500 cm^−1^, spectral resolution of 4 cm^−1^, and 32 consecutive scans. All spectra were recorded in transmittance mode for subsequent structural analysis.

#### 2.4.3. Scanning Electron Microscopy (SEM) Observation

The morphological characteristics were observed with reference to the method of Rani S R [27], with slight modifications. The *Apocynum venetum* protein and ACE-inhibitory peptide samples were freeze-dried and then mounted onto sample stubs, followed by gold sputter coating.

The microstructure was examined under a scanning electron microscope at an accelerating voltage of 5 kV, with magnifications of 500×, 1000×, and 5000×. SEM images were recorded to analyze the microstructural morphology of the peptides.

#### 2.4.4. Ultraviolet–Visible (UV–Vis) Absorption Spectroscopy Analysis

UV–Vis absorption spectra were measured with reference to the method of Yang Chengjun [28], with minor modifications. *Apocynum venetum* protein and ACE-inhibitory peptide samples were dissolved in phosphate-buffered saline (PBS) to prepare solutions at concentrations ranging from 0.05 to 0.5 mg/mL. PBS was used as the blank control. The scanning wavelength range was set from 220 to 400 nm, with a slit width of 1.0 mm and a sampling interval of 0.5 nm.

#### 2.4.5. Determination of Free Amino Acid Content

The content of 17 free amino acids was determined using high-performance liquid chromatography (HPLC), with reference to the method of Zhao Chenfei [29], with slight modifications. A C18 reversed-phase column (250 mm × 4.6 mm, 5 μm) was used for separation. The mobile phase consisted of solvent A (methanol–acetonitrile, 20:80, *v*/*v*) and solvent B (0.05 mol/L sodium acetate–acetonitrile mixture), with gradient elution. The flow rate was set at 0.8 mL/min, the detection wavelength was 254 nm, the column temperature was maintained at 30 °C, and the injection volume was 10 μL. Samples were derivatized using 0.1 mol/L triethylamine in acetonitrile and 0.1 mol/L phenyl isothiocyanate in acetonitrile, followed by extraction with n-hexane and membrane filtration prior to analysis. Standard solutions of 17 amino acids were prepared through derivatization and serial dilution of a mixed standard. A calibration curve was established using concentration as the x-axis and peak area as the y-axis, and amino acid contents in samples were calculated based on the corresponding regression equations.

### 2.5. Isolation and Screening of Peptides

The peptide samples were desalted and purified using Ziptip C18 solid-phase extraction tips (Millipore, Billerica, MA, USA), and 1 μL of the purified sample was injected for analysis. Separation was performed using a Thermo Scientific Vanquish Neo UHPLC system. Mobile phase A consisted of 0.1% formic acid in water, while mobile phase B consisted of 80% acetonitrile. Chromatographic separation was carried out using an analytical column coupled with a pre-column. Subsequently, peptide analysis was conducted on a Thermo Scientific Orbitrap Astral high-resolution mass spectrometer equipped with a nano-electrospray ionization (nanoESI) source. Data were acquired in data-independent acquisition (DIA) mode. The raw data were processed using Spectronaut 20.1 software (Biognosys). Peptidomic analysis was performed based on a directDIA library-free search strategy, a FASTA protein sequence database, and unspecific enzymatic cleavage parameters.

#### 2.5.1. Peptide Screening

Based on the peptide sequences identified from LC–MS/MS analysis of *Apocynum venetum* protein hydrolysates, the PeptideRanker online tool was employed to predict bioactivity. Peptides with a PeptideRanker score ≥ 0.8 were selected as candidate bioactive peptides [30]. Subsequently, the physicochemical properties of the selected peptides were analyzed using the ProtParam tool (https://web.expasy.org/protparam/, accessed 25 November 2025). Screening criteria included a hydrophobicity value > 0 (to ensure membrane permeability) and an isoelectric point (pI) between 6 and 8 (to match physiological pH conditions and maintain stability) [31]. The resulting target peptides were further subjected to molecular docking with angiotensin-converting enzyme (ACE) using the HPEPDOCK 2.0 software to investigate their binding modes and binding affinities with the active site of ACE, thereby evaluating their potential inhibitory activity.

#### 2.5.2. Molecular Docking Method

Molecular docking was performed using the HPEPDOCK 2.0 online server (http://huanglab.phys.hust.edu.cn/hpepdock/, accessed 25 November 2025). The crystal structure of ACE (PDB ID: 1O8A) was downloaded from the Protein Data Bank (http://www.rcsb.org) [32]. The structure was preprocessed using PyMOL (Version 2.6.x) software by removing crystallographic water molecules, small-molecule ligands, and redundant ions, while retaining the complete protein backbone and side chains. Hydrogen atoms were added to the receptor structure, which was then used as the ACE receptor model. The optimized three-dimensional structures of the target peptides were constructed and used as ligands. Both receptor and ligand structures were uploaded to the HPEPDOCK 2.0 server for fully automated docking. According to the default parameters of the server and commonly used literature thresholds, docking scores ≤ −200 were defined as effective binding criteria. The optimal binding conformations were selected for further analysis of binding interactions and active site residues. Finally, the docking results were visualized in three dimensions using PyMOL software.

### 2.6. Statistical Analysis

All experiments were performed in triplicate, and data were expressed as mean ± standard deviation. One-way analysis of variance (ANOVA) and response surface regression analysis were performed using SPSS 26.0 software. Experimental design and data fitting for response surface methodology were conducted using Design-Expert 12.0 software. Statistical significance was defined as *p* < 0.05, and highly significant differences were defined as *p* < 0.01. Structural characterization data were analyzed and plotted using instrument-specific software and Origin 2021. LC–MS/MS data were processed using PeakView software (Version 2.2) for peptide identification and molecular weight analysis. Molecular docking results were analyzed using HPEPDOCK 2.0 and PyMOL software. Binding energy values were expressed as mean ± standard deviation and statistically analyzed using SPSS software.

## 3. Results and Discussion

### 3.1. Extraction of Apocynum venetum Protein and Selection of Protease

**Figure 3:**
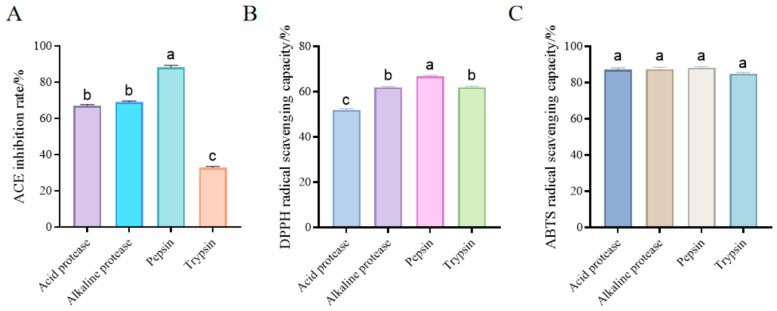
Effects of different proteases on ACE inhibition rate and antioxidant capacity of hydrolysates. (**A**): ACE inhibition rate; (**B**): DPPH radical scavenging capacity; (**C**): ABTS radical scavenging capacity. Values are presented as mean ± standard deviation (n = 3). Different lowercase letters (a, b, c) above the bars indicate significant differences among groups (*p* < 0.05).

As shown in Figure 3, using ACE inhibitory activity as the primary evaluation index and antioxidant capacity as the secondary index, pepsin exhibited the best overall performance among the four tested proteases. The ACE inhibitory rate of pepsin hydrolysates was approximately 88% Figure 3A, which was significantly higher than that of alkaline protease (~69%), acidic protease (~67%), and trypsin (~33%). In addition, pepsin hydrolysates also showed the strongest antioxidant activities, with ABTS radical scavenging activity reaching approximately 87% (Figure 3C) and DPPH radical scavenging activity reaching approximately 65% (Figure 3B). This superior performance can be attributed to the fact that pepsin preferentially hydrolyzes peptide bonds adjacent to aromatic and hydrophobic amino acid residues under acidic conditions [33], thereby efficiently releasing short peptides from *Apocynum venetum* proteins that are more likely to interact with the active site of ACE. The resulting hydrolysates are mainly low-molecular-weight peptides, which are conducive to enhancing ACE inhibitory activity while simultaneously exposing more antioxidant functional groups. Moreover, the acidic hydrolysis environment of pepsin closely mimics physiological gastric conditions, which further supports its suitability for generating bioactive peptides. Therefore, pepsin was selected as the optimal protease for the preparation of ACE-inhibitory peptides from *Apocynum venetum*. These findings are consistent with those reported by Ngo D et al. [34].

### 3.2. Results and Analysis of Single-Factor Experiments

Using pepsin as the hydrolytic enzyme, the effects of substrate concentration, enzyme dosage, and hydrolysis time on the ACE inhibitory activity of *Apocynum venetum* protein hydrolysates were systematically investigated.

**Figure 4:**
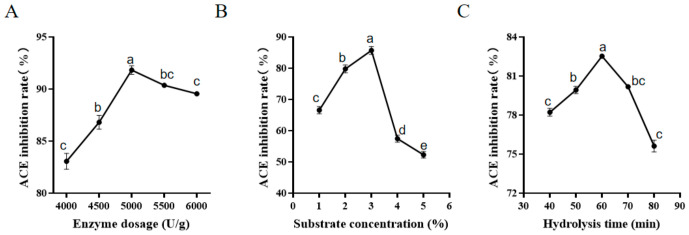
Results of Single-Factor Experiments. (**A**) Substrate concentration; (**B**) Enzyme dosage; (**C**) Hydrolysis time. All data were analyzed via one-way ANOVA followed by Duncan’s multiple range test. Different lowercase letters within one subfigure indicate significant differences at *p* < 0.05.

As shown in Figure 4A, the ACE inhibitory activity initially increased and then decreased with increasing substrate concentration, reaching a maximum value of 86.51% at 3%. This trend can be explained by the fact that at low substrate concentrations, enzyme–substrate interactions are sufficient, facilitating the release of bioactive peptides. However, excessively high substrate concentrations increase system viscosity and steric hindrance, thereby reducing enzymatic efficiency and interfering with the interaction between peptides and ACE [35].

Figure 4B shows that ACE inhibitory activity first increased and then decreased with increasing enzyme dosage, reaching a maximum of 92.16% at 5000 U/g. Insufficient enzyme dosage leads to incomplete hydrolysis, while excessive enzyme levels may result in over-hydrolysis of proteins, causing partial degradation of ACE-inhibitory peptides into inactive small molecules. In addition, the accumulation of non-functional peptides may also weaken inhibitory activity [36].

Hydrolysis time significantly influences the degree of protein hydrolysis and peptide chain length, thereby affecting the bioactivity of peptides [37]. As shown in Figure 4C, ACE inhibitory activity initially increased and then decreased with increasing hydrolysis time, reaching a maximum of 82.44% at 60 min. Beyond this point, activity decreased significantly. This is because bioactive peptides are continuously released and accumulated during the initial hydrolysis stage, whereas prolonged hydrolysis may disrupt key structural domains of active peptides, resulting in the loss of their ability to bind to the active site of ACE [38].

### 3.3. Response Surface Experimental Results and Analysis

**Figure 5:**
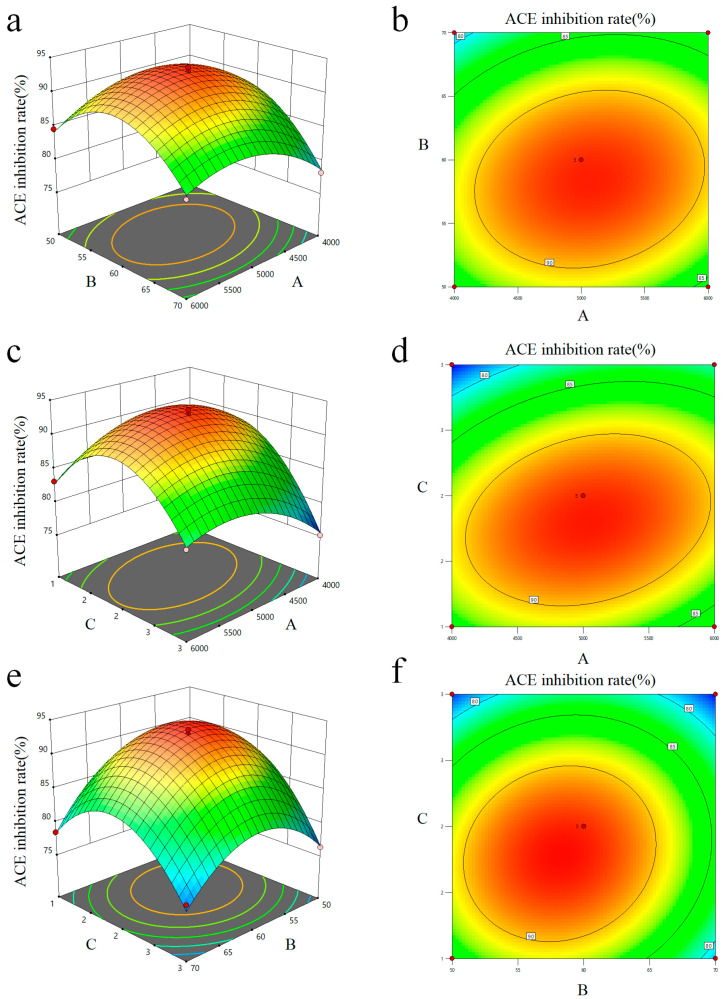
Response Surface and Contour Plots Showing the Effects of Factor Interactions on ACE Inhibitory Activity. Note: Substrate concentration (C); hydrolysis time (B); enzyme dosage (A). (**a**) 3D response surface plot for the interaction between enzyme dosage (A) and hydrolysis time (B); (**b**) 2D contour plot for the interaction between enzyme dosage (A) and hydrolysis time (B); (**c**) 3D response surface plot for the interaction between enzyme dosage (A) and substrate concentration (C); (**d**) 2D contour plot for the interaction between enzyme dosage (A) and substrate concentration (C); (**e**) 3D response surface plot for the interaction between hydrolysis time (B) and substrate concentration (C); (**f**) 2D contour plot for the interaction between hydrolysis time (B) and substrate concentration (C).

The response surface plot and contour plots illustrating the effects of interaction among various factors on the ACE inhibition rate of *Apocynum venetum* are shown in Figure 5. ANOVA results (Table 3) showed that the model was highly significant (F = 115.90, *p* < 0.0001), with no significant lack of fit (*p* = 0.1139), indicating good agreement between the model and experimental data. The model exhibited strong reliability, with R^2^ = 0.9933, adjusted R^2^ = 0.9848, and predicted R^2^ = 0.9182. The CV was 0.9461% and the Adeq Precision was 28.39, confirming high precision and a strong signal-to-noise ratio.

Factor effects followed the order: C > B > A. Substrate concentration (C), hydrolysis time (B), all interaction terms, and quadratic terms were highly significant (*p* < 0.01), while enzyme dosage (A) was significant (*p* < 0.05). The positive coefficient of A indicates a promoting effect, whereas negative coefficients for B and C suggest that excessive hydrolysis or substrate concentration reduces activity. Significant positive interaction terms, especially AC, indicate strong synergistic effects. The quadratic terms confirm a clear optimal region.

The second-order polynomial regression equation was established as:

Y = 92.87 + 0.7050A − 2.01B − 2.89C + 1.36AB + 2.29AC + 1.48BC − 3.78A^2^ − 6.34B^2^ − 7.65C^2^ (2)

The response surface results are shown in Table 4. Validation under optimized conditions (A = 4997.89 U/g, B = 58.17 min, C = 1.79%) yielded an experimental ACE inhibitory rate of 92.34%, close to the predicted value (93.35%) with a deviation of 1.08%, confirming the model’s accuracy and reliability.

### 3.4. Structural Characterization Results and Analysis

#### 3.4.1. Endogenous Fluorescence Spectra

**Figure 6:**
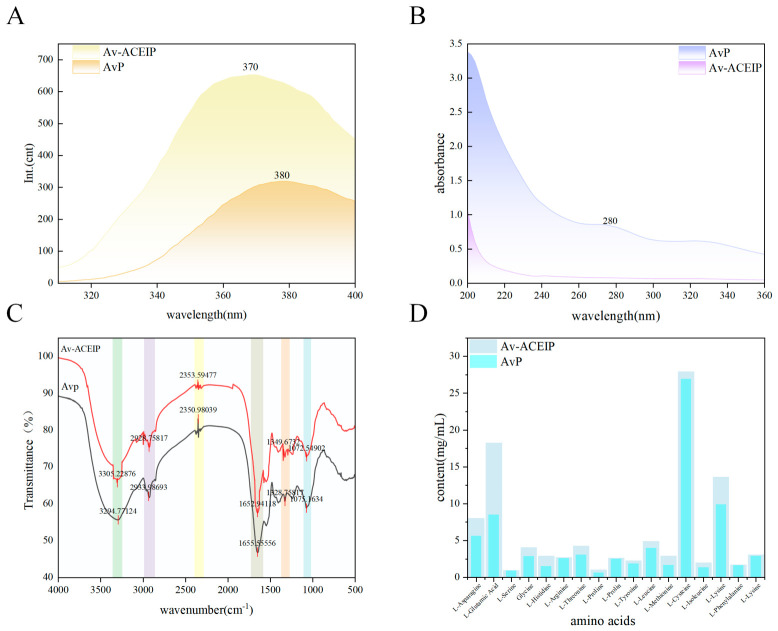
Structural characterization of *Apocynum venetum* protein and peptide. (**A**) Endogenous fluorescence spectra of protein/peptide; (**B**) UV absorption spectra of protein/peptide; (**C**) Fourier transform infrared (FTIR) spectra of protein/peptide; (**D**) Amino acid composition of protein/peptide.

As shown in Figure 6A, the maximum emission wavelength of *Apocynum venetum* protein (AvP) was observed at 380 nm, whereas that of the pepsin-derived ACE-inhibitory peptides (Av-ACEIP) exhibited a blue shift to 370 nm, accompanied by a marked increase in fluorescence intensity. The blue shift indicates that aromatic fluorophores such as tryptophan and tyrosine residues are transferred from a polar aqueous environment to a more hydrophobic microenvironment, suggesting that enzymatic hydrolysis disrupted the compact higher-order structure of AvP and exposed previously buried hydrophobic groups [39]. The increased fluorescence intensity further confirms that aromatic residues, previously quenched in the native protein conformation, are released due to peptide unfolding, thereby enhancing fluorescence emission.

It has been reported that the S1′ pocket of ACE exhibits strong hydrophobic characteristics and shows preferential affinity toward peptides containing hydrophobic or aromatic side chains [40]. The abundant exposed hydrophobic and aromatic residues endow Av-ACEIP with favorable structural prerequisites for binding to ACE, which theoretically benefits hydrophobic association with the ACE pocket. However, spectral conformational changes alone cannot verify the real binding interaction between peptides and the ACE active site. The accurate binding mode, intermolecular forces and stable complex formation between screened peptides and ACE are further validated and characterized by the subsequent in silico molecular docking analysis. This favorable structural property implied by fluorescence spectra lays a potential structural foundation for the potent ACE inhibitory and hypotensive capacity of Av-ACEIP.

#### 3.4.2. UV–Visible Absorption Spectroscopy Analysis

As shown in Figure 6B, Apocynum venetum protein (AvP) exhibited a characteristic absorption peak at 280 nm, which is attributed to the π→π* electronic transitions of aromatic amino acid residues such as tryptophan and tyrosine [41]. In contrast, this distinct 280 nm absorption peak was absent in pepsin-hydrolyzed ACE-inhibitory peptides (Av-ACEIP), demonstrating that enzymatic hydrolysis substantially changed the abundance and microenvironment distribution of aromatic residues.

In the native folded conformation of AvP, aromatic amino acid residues are mostly embedded inside the protein’s hydrophobic core [42], which accounts for the obvious UV absorption signal at 280 nm. Pepsin digestion breaks the compact tertiary structure of intact protein, generating short peptide fragments; this process rearranges or releases aromatic residue-containing segments, thus greatly altering the exposure status and relative content of aromatic residues in Av-ACEIP [43].

Both AvP and Av-ACEIP displayed intense absorption signals ranging from 200 to 220 nm, originating from n→π* electronic transitions of peptide bonds. This result verifies that the peptide backbone skeleton remains complete after enzymatic hydrolysis. The vanishing of the 280 nm aromatic residue absorption peak, combined with maintained strong absorbance at 200–220 nm, collectively reveals that Av-ACEIP mainly consists of low-molecular-weight peptides with modified aromatic residue microenvironment and content.

The above conformational differences reflected by UV-vis spectra indicate that Av-ACEIP possesses favorable structural characteristics that are theoretically conducive to binding the hydrophobic ACE pocket. Nevertheless, UV spectral data alone cannot confirm direct binding interactions between Av-ACEIP and the ACE active site. The precise binding mode and intermolecular interactions between candidate peptides and ACE are further validated via subsequent in silico molecular docking analysis. Such structural variations observed from UV spectra only provide potential spectroscopic clues to explain the superior ACE-inhibitory capacity of Av-ACEIP.

#### 3.4.3. FT-IR Analysis

The Fourier transform infrared (FTIR) spectra of *Apocynum venetum* protein (AvP) and its hydrolysates (Av-ACEIP) are shown in Figure 6C. Significant changes in the amide bands after enzymatic hydrolysis indicate protein degradation and the formation of ACE-inhibitory peptides.

The amide A band (3305–3294 cm^−1^, N–H stretching vibration) shifted from 3294.77 cm^−1^ in AvP to 3305.23 cm^−1^ in Av-ACEIP, accompanied by an increase in transmittance. This blue shift suggests that enzymatic hydrolysis disrupted intermolecular hydrogen bonding networks, exposing N–H groups and providing a more flexible conformational basis for peptide fragments [44].

The amide I band (1655–1652 cm^−1^, C=O stretching vibration) shifted from 1655.56 cm^−1^ in AvP to 1652.94 cm^−1^ in Av-ACEIP, with a notable increase in transmittance. This indicates that enzymatic hydrolysis disrupted ordered secondary structures such as α-helices and β-sheets, leading to the formation of small peptides predominantly in random coil conformation [45]. Such increased structural flexibility facilitates better adaptation to the ACE active site, forming the structural basis for inhibitory activity.

Changes in the amide III band (1349–1328 cm^−1^, C–N stretching and N–H bending vibrations) further confirm efficient peptide bond cleavage and the formation of short-chain ACE-inhibitory peptides. In addition, the blue shift of the amide B band (2933–2928 cm^−1^, C–H stretching vibration) reflects exposure of hydrophobic side chains, which may enhance membrane permeability.

Overall, enzymatic hydrolysis transformed the protein secondary structure into a predominantly random coil conformation. The resulting peptides, with improved conformational flexibility and exposed hydrophobicity, provide favorable structural characteristics for efficient binding to ACE and exerting antihypertensive activity.

#### 3.4.4. Amino Acid Composition Analysis

The comparison of 17 amino acid contents in *Apocynum venetum* protein (AvP) and its hydrolysates (Av-ACEIP) is shown in Figure 6D. After enzymatic hydrolysis, most free amino acids exhibited a significant increase, with L-glutamic acid, L-aspartic acid, and L-lysine showing particularly pronounced enrichment [46]. This is attributed to the efficient cleavage of peptide bonds during hydrolysis, which converts long-chain proteins into short peptides and releases a large amount of free amino acids.

From the perspective of structure–activity relationships of ACE-inhibitory peptides, acidic amino acids (aspartic acid and glutamic acid) can enhance binding affinity to positively charged residues in the ACE active site via electrostatic interactions [47]. Basic amino acids such as lysine may stabilize peptide–enzyme complexes through hydrogen bonding and improve peptide solubility and bioavailability. Hydrophobic amino acids further provide key interaction interfaces for peptide insertion into the hydrophobic pocket of ACE.

These free amino acids not only serve as fundamental building blocks of ACE-inhibitory peptides but may also synergistically enhance overall antihypertensive activity. In addition, their enrichment provides a rich amino acid basis for the subsequent screening of highly active ACE-inhibitory peptides, confirming that enzymatic hydrolysis effectively enriches both bioactive peptide fragments and functional amino acid components with antihypertensive potential.

#### 3.4.5. SEM Analysis

**Figure 7:**
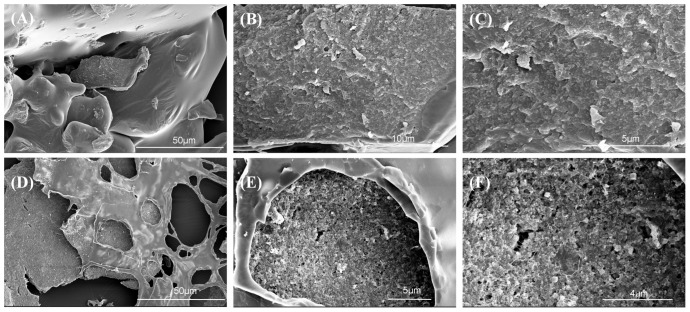
Scanning electron microscopy (SEM) images of *Apocynum venetum* protein and peptide. Note: (**A**–**C**) *Apocynum venetum* protein before enzymatic hydrolysis at magnifications of 1000×, 5000×, and 10,000×, respectively; (**D**–**F**) freeze-dried ACE-inhibitory peptides obtained after pepsin hydrolysis at magnifications of 100×, 5000×, and 10,000×, respectively.

As shown in Figure 7, *Apocynum venetum* protein (A–C) exhibited a dense, continuous aggregated structure with relatively smooth surfaces. The molecules were tightly packed through hydrogen bonding and hydrophobic interactions, consistent with the typical microstructure of native macromolecular proteins.

After pepsin hydrolysis, the freeze-dried ACE-inhibitory peptides (D–F) showed a completely disrupted structure, transforming into a loose, porous, and highly fragmented honeycomb-like morphology with a markedly reduced particle size. Protein hydrolysis and partial denaturation typically induce surface collapse and rupture [48]. It has been reported that such porous protein structures can enhance enzymatic accessibility and improve ACE inhibitory activity [49].

This morphological transformation is attributed to pepsin-specific cleavage of peptide bonds, which breaks long-chain proteins into short peptides, disrupts secondary and tertiary structures, weakens intermolecular forces, and prevents the maintenance of the original compact aggregates, ultimately resulting in a porous microstructure [50].

### 3.5. Peptide Screening and Molecular Docking

#### 3.5.1. LC–MS/MS Separation and Peptide Screening

**Figure 8:**
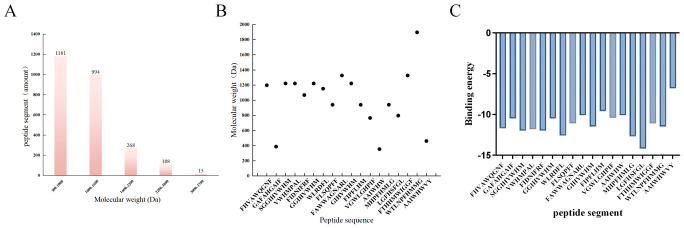
Peptide molecular weight distribution of *Apocynum venetum* hydrolysates. (**A**) Molecular weight distribution of peptides derived from *Apocynum venetum* protein hydrolysates. (**B**) Molecular weight distribution of 18 selected peptide sequences. (**C**) Binding Affinity of 18 Peptide Segments via Molecular Docking.

Previous studies have shown that ACE-inhibitory peptides are typically low-molecular-weight peptides with molecular weights below 3 kDa [51]. As shown in Figure 8A, the molecular weight distribution of *Apocynum venetum* protein hydrolysates was mainly concentrated in the range of 500–1600 Da, with the 500–1000 Da fraction being the most abundant (46.0%). This profile is consistent with the typical molecular characteristics of ACE-inhibitory peptides.

The total ion chromatogram (TIC) from LC–MS/MS analysis indicated good chromatographic separation of the enzymatic hydrolysates within 0–12.5 min, a total of 2567 peptide sequences were identified, demonstrating high peptide diversity. This provides a rich candidate pool for subsequent screening and functional validation of highly active ACE-inhibitory peptides.

#### 3.5.2. Molecular Docking

A total of 18 specific peptides were identified from *Apocynum venetum* protein hydrolysates using LC–MS/MS. As shown in Figure 8B, their molecular weights ranged from 400 to 1900 Da, with most peptides distributed between 800 and 1300 Da. This molecular weight profile is consistent with the typical characteristics of bioactive peptides, which not only favor interaction with the ACE active site but also support potential intestinal absorption.

Among the identified peptides, GAFAHGAIF was the smallest peptide (approximately 380 Da), while WTLNPFHMMG was the largest (approximately 1900 Da), indicating a wide molecular size distribution. This diversity provides a structural basis for screening peptides with different binding behaviors and biological activities.

As shown in the bar chart of peptide binding energies (Figure 8C), all peptide segments can bind spontaneously to the target protein, but there are significant differences in binding affinity; a lower binding energy value indicates higher stability of the peptide-protein complex. The binding energies of most peptide segments are concentrated in the range of −10 to −12 kcal/mol, and only a few short peptides exhibit high binding potential. This study ultimately identified the hexapeptide WLRDFL as the target active peptide for the following key reasons: First, WLRDFL has the lowest binding energy among all tested peptide segments; thermodynamically, it exhibits the strongest binding affinity to the target protein, with optimal synergistic interactions between intermolecular hydrophobic forces, π–π stacking, and hydrogen bonds; Second, as a short hexapeptide, it lacks the steric hindrance associated with longer peptides. Its flexible conformation allows it to fully embed into the protein binding pocket, while the sufficient number of residues provides multiple molecular interaction sites, balancing binding affinity and interaction strength; Third, the sequence contains two types of key aromatic hydrophobic residues—tryptophan (W) and phenylalanine (F)—which form the structural basis for its extremely low binding energy. Compared to other peptide segments that lack aromatic residues, have excessively long peptide chains, or are enriched with hydrophilic residues—resulting in higher binding energies—WLRDFL possesses significant advantages in both binding stability and spatial fit; therefore, this peptide was selected.

The analysis of PeptideRanker activity scores, isoelectric points, and hydrophobicity of the 18 peptides is summarized in Table 5. The PeptideRanker scores ranged from 0.80 to 0.97, indicating generally high predicted bioactivity. Among them, FLSQPFF (0.97), FIDNIFRF (0.96), and WLRDFL (0.95) ranked among the top candidates, suggesting strong potential biological activity.

The isoelectric points ranged from 5.52 to 9.75, with most peptides clustering near neutrality (6.7–6.9). This distribution indicates good compatibility with physiological pH conditions, which helps maintain peptide structural stability and solubility in biological systems.

Hydrophobicity values ranged from 0.18 to 1.57. Peptides such as FHVAWQGNF and WTLNPFHMMG exhibited relatively higher hydrophobicity, which may facilitate interactions with the hydrophobic pocket of ACE. In contrast, peptides with moderate hydrophobicity may contribute to more sustained bioactivity in vivo due to improved balance between solubility and membrane interaction.

**Figure 9:**
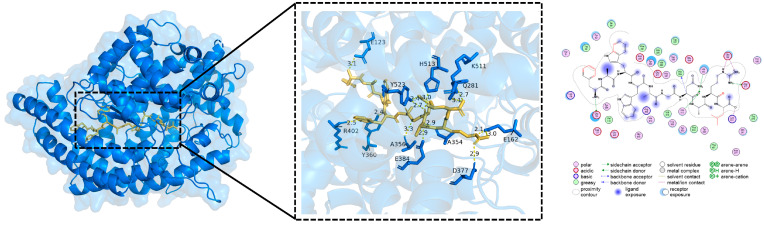
Three-dimensional molecular docking model of peptide WLRDFL with ACE.

Figure 9 further illustrates the molecular docking conformation of the peptide WLRDFL with ACE, highlighting its interaction mode within the active site.

Molecular docking results further validated the binding affinity and interaction modes between the candidate peptides and ACE. All screened peptides were able to stably bind within the ACE active-site pocket, showing a high degree of overlap with the binding region of the natural substrate, which suggests a potential competitive inhibition mechanism.

In terms of molecular interactions, the predominant peptides mainly interacted with key residues in the ACE active site through hydrogen bonding, hydrophobic interactions, and electrostatic forces. Previous studies have reported that the ACE active site consists of three functional subsites: the S1 pocket (Ala354, Glu384, Tyr523), the S2 pocket (Gln281, His353, Lys511, His513, Tyr520), and the S1′ pocket (Glu162) [52].

As shown in Table 6 and Figure 9, among the peptides derived from *Apocynum venetum*, WLRDFL (binding energy: −12.6 kcal/mol) was the only peptide that simultaneously targeted all three subsites (S1, S2, and S1′). Its hydrogen-bonding interactions comprehensively covered key catalytic residues of ACE, indicating the most stable binding conformation and the strongest inhibitory potential.

Strong-binding peptides such as MHPFHMLG (−12.7 kcal/mol) and FIDNIFRF (−12.0 kcal/mol), as well as moderately strong binders such as FHVAWQGNF (−11.7 kcal/mol) and VWHMPAL (−11.8 kcal/mol), mainly interacted with both S1 and S2 pockets, forming stable hydrogen-bond networks and demonstrating good competitive inhibition potential. In contrast, weak-binding peptides such as SGGIHVVWHM and VGWLGHPIF interacted with only a single pocket or lacked binding to core catalytic regions, with binding energies generally lower than −11.0 kcal/mol, indicating limited inhibitory potential.

Overall, a positive correlation was observed between peptide–ACE binding affinity and active-site matching degree. Among all candidates, WLRDFL showed the most comprehensive interaction profile and the strongest binding affinity, suggesting that it could serve as a promising lead peptide for the development of antihypertensive functional agents.

## 4. Discussion

### 4.1. Optimization of Extraction and Enzymatic Hydrolysis

Protein from *Apocynum venetum* was successfully extracted using an alkali extraction–acid precipitation method. Among the four proteases evaluated, pepsin exhibited the highest ACE inhibitory activity, indicating its superior efficiency in releasing bioactive peptides enriched in hydrophobic residues under acidic conditions.

Response surface methodology further demonstrated that substrate concentration, enzyme dosage, and hydrolysis time significantly influenced ACE inhibitory activity, all showing a typical non-linear response. The optimal hydrolysis conditions were determined as follows: enzyme dosage of 4997.89 U/g, hydrolysis time of 58.17 min, and substrate concentration of 1.79%. Under these conditions, the ACE inhibitory rate reached 92.34%.

Compared with previously reported plant-derived ACE inhibitory peptides, the present process offers advantages in terms of mild reaction conditions, short processing time, and high inhibitory efficiency, suggesting strong potential for industrial-scale application. Further improvements may be achieved by integrating assisted hydrolysis technologies (e.g., ultrasound or multi-enzyme systems) to enhance peptide yield and bioactivity.

### 4.2. Structural Characteristics of ACE Inhibitory Peptides

Spectroscopic analyses confirmed that enzymatic hydrolysis significantly altered the structural properties of *Apocynum venetum*-derived peptides. Fluorescence spectroscopy showed a blue shift accompanied by increased intensity, indicating exposure of aromatic residues (Trp, Tyr) and a transition to a more hydrophobic microenvironment. This structural rearrangement enhances peptide affinity toward the hydrophobic pockets of ACE.

FT-IR analysis revealed marked shifts in amide bands, suggesting disruption of ordered secondary structures and conversion into random coil-dominated conformations, which are more flexible and favorable for enzyme binding. UV absorption at 280 nm was significantly reduced after hydrolysis, indicating changes in aromatic amino acid environments and peptide chain fragmentation.

In addition, amino acid composition analysis showed a notable increase in acidic, basic, and hydrophobic residues. These residues contribute synergistically to ACE inhibition through electrostatic interactions, hydrogen bonding, and hydrophobic forces.

SEM observations further demonstrated that compact protein aggregates were transformed into porous and fragmented microstructures after enzymatic hydrolysis, which is consistent with enhanced accessibility and bioactivity.

### 4.3. Peptide Identification and Molecular Docking Analysis

A total of 2567 peptides were identified by LC-MS/MS, among which 18 candidate peptides (400–1900 Da) were selected based on bioinformatics screening. These peptides exhibited high PeptideRanker scores, moderate hydrophobicity, and physiological pI values, indicating strong potential bioactivity and good compatibility with physiological conditions.

Molecular docking analysis revealed that all candidate peptides could stably bind to the active site of ACE mainly through hydrogen bonding and hydrophobic interactions, suggesting a competitive inhibition mechanism.

Notably, peptide WLRDFL exhibited the strongest binding affinity (−12.6 kcal/mol) and simultaneously interacted with the S1, S2, and S1′ pockets of ACE, forming an extensive hydrogen bond network across the catalytic region. This multi-site binding mode suggests a highly stable enzyme–peptide complex and strong inhibitory potential.

Other peptides such as MHPFHMLG and FIDNIFRF also showed strong binding affinities, mainly targeting dual-pocket interactions. These findings are consistent with previous reports that potent ACE inhibitory peptides often possess multi-residue interactions within the catalytic core.

### 4.4. Limitations and Future Perspectives

Although this study systematically investigated peptide preparation, structural characterization, and in silico analysis, several limitations remain.

First, biological validation at the cellular and in vivo levels (e.g., vascular endothelial cells and hypertensive animal models) was not performed. Second, peptide stability during gastrointestinal digestion and absorption mechanisms were not evaluated. Third, purification, IC50 determination, and functional stability assays were not conducted.

Future studies should focus on peptide synthesis and functional validation of key sequences such as WLRDFL, followed by gastrointestinal stability and intestinal transport assays. In vivo antihypertensive evaluation and omics-based mechanism studies are also necessary to elucidate regulatory pathways. Additionally, formulation development (e.g., microencapsulation or nano-delivery systems) may improve peptide bioavailability and enable functional food applications.

## 5. Conclusions

This study systematically investigated the preparation, optimization, structural characterization, peptide identification, and molecular interaction mechanisms of ACE inhibitory peptides derived from *Apocynum venetum*.

Pepsin was identified as the most efficient enzyme for producing bioactive peptides, and optimized hydrolysis conditions yielded a high ACE inhibitory activity of 92.34%. Structural analyses confirmed that enzymatic hydrolysis transformed native proteins into flexible, small-molecule peptides enriched in exposed hydrophobic and bioactive amino acid residues.

LC-MS/MS and bioinformatics screening identified 18 candidate peptides, among which WLRDFL demonstrated the strongest binding affinity and multi-pocket interaction with ACE, suggesting its potential as a lead antihypertensive peptide.

This study has so far screened ACE-inhibitory peptides from *Apocynum venetum* based solely on in vitro enzyme activity assays and molecular docking. Its primary application focus is the development of dietary supplements and functional food ingredients, rather than the direct development of peptide drugs for clinical treatment. Future research will proceed along two tracks: first, conducting activity validation at the cellular level to evaluate the effects of the highly active peptide WLRDFL on the ACE pathway in vascular endothelial cells; second, evaluating its antihypertensive efficacy in vivo using animal models of hypertension. If in vivo activity proves to be excellent, WLRDFL could serve as a lead scaffold for a peptide therapeutic, allowing for further molecular modification and optimization of drug candidates. Overall, this work provides new insights into the development of natural ACE inhibitory peptides and highlights the high-value utilization potential of *Apocynum venetum* as a functional food resource.

## Figures and Tables

**Table 1:** Optimal enzymatic hydrolysis conditions for different proteases.

Protease	Optimal Temperature (°C)	Optimal pH
Pepsin	37	2
Trypsin	37	8
Acid Protease	45	2.5
Alkaline Protease	55	10

**Table 2:** Experimental Procedure for Determination of ACE Inhibitory Activity.

Components	Sample A (μL)	Control B (μL)	Blank C (μL)
Substrate solution (6.5 mmol/L HHL)	200	200	200
Boric acid buffer (0.1 mol/L pH 8.3)	0	0	80
Peptide solution	80	80	
Mixed thoroughly and incubated in a water bath at 37 °C for 5 min.			
ACE (0.1 UN/mL)	20	0	20
Boric acid buffer (0.1 mol/L pH 8.3)	0	20	0
Mixed thoroughly and incubated in a water bath at 37 °C for 5 min.			
HCl (1 mol/L)	250	250	250
Vortexing, Let stand for 5 min			
Ethyl acetate	1700	1700	1700

**Table 3:** Analysis of Variance Table.

Source of Variation	Sum of Squares	Degrees of Freedom	Mean Square	F-Value	*p*-Value	Significance
Model	666.76	9	74.08	115.90	<0.0001	***
A-Enzyme dosage	3.98	1	3.98	6.22	0.0413	*
B-Hydrolysis time	32.28	1	32.28	50.50	0.0002	**
C-Substrate concentration	66.99	1	66.99	104.80	<0.0001	***
AB	7.45	1	7.45	11.66	0.0112	*
AC	21.07	1	21.07	32.96	0.0007	**
BC	8.79	1	8.79	13.75	0.0076	*
A^2^	60.01	1	60.01	93.88	<0.0001	***
B^2^	169.12	1	169.12	264.58	<0.0001	***
C^2^	246.59	1	246.59	385.77	<0.0001	***
Residual	4.47	7	0.6392	-	-	-
Lack of Fit	3.32	3	1.11	3.83	0.1139	
Pure Error	1.16	4	0.2891	-	-	-
Cor Total	671.23	16	-	-	-	-
R^2^	0.9933					
Radj^2^	0.9848					

Note: * indicates a significant difference (*p* < 0.05), ** indicates a moderately significant difference (*p* < 0.01), and *** indicates an extremely significant difference (*p* < 0.0001).

**Table 4:** Experimental design and results of Box–Behnken Design (BBD).

Test Number	A-Enzyme Addition Amount (U)	B-Enzymatic Hydrolysis Time (min)	C-Bottom Concentration (%)	ACE Suppression Rate (%)
1	4000	70	2	78.19
2	5000	70	1	78.45
3	5000	70	3	76.35
4	5000	50	1	84.37
5	5000	60	2	92.37
6	6000	60	3	81.23
7	4000	50	2	86
8	5000	50	3	76.34
9	6000	60	1	83.15
10	5000	60	2	92.35
11	4000	60	1	86.24
12	6000	70	2	82.24
13	6000	50	2	84.59
14	5000	60	2	92.96
15	5000	60	2	93.01
16	4000	60	3	75.14
17	5000	60	2	93.65

**Table 5:** Bioactivity scores, hydrophobicity, and isoelectric points of 18 peptide sequences.

Peptide Sequence	PeptideRanker Activity	Isoelectric Points	Hydrophobicity
FHVAWQGNF	0.94	6.74	1.57
GAFAHGAIF	0.93	6.74	0.30
SGGIHVWHM	0.91	6.79	0.52
VWHMPAL	0.90	7.33	0.85
FIDNIFRF	0.96	5.84	0.74
GGIHVWHM	0.85	6.96	0.58
WLRDFL	0.95	5.84	0.25
FLSQPFF	0.97	5.52	0.9
FAWWAGNARL	0.95	9.75	0.18
GIHVWHM	0.83	6.71	0.86
FIPFLHM	0.83	6.89	1.02
VGWLGHPIF	0.83	6.78	0.75
AAIWHW	0.82	6.74	0.79
MHPFHMLG	0.82	6.92	0.69
LGFHSFGL	0.82	6.92	0.69
FTHHMWIGGF	0.82	6.92	0.69
WTLNPFHMMG	0.80	6.74	1.28
AAIWHWVY	0.82	6.74	1.05

**Table 6:** Molecular docking results of peptide–ACE interactions (PDB ID: 1O8A).

Peptide Sequence	Binding Energy (kcal/mol)	Number of H-Bonds	Bond Length (Å)	Hydrogen-Bonded Residues
FHVAWQGNF	−11.7	9	2.8, 2.5, 3.0, 2.4, 2.2, 3.3, 2.6, 3.1	Y360, A356, R124, Y135, R522, Y523, H513
GAFAHGAIF	−10.5	6	1.8, 2.4, 2.8, 3.0, 3.3, 3.4	Y62, N66, R124, R522
SGGIHVWHM	−12.0	6	2.4, 2.7, 3.1, 3.2, 3.3, 3.4	D415, Y523, R124
VWHMPAL	−11.8	5	2.8, 3.3, 3.3	H512, Y523, H353, A354, Y360
FIDNIFRF	−12.0	7	1.1, 2.2, 2.3, 2.6, 3.0, 3.1, 3.3, 3.4	R522, S219, E123, D121, Y360
GGIHVWHM	−10.5	5	2.4, 2.9, 3.1, 3.3, 3.4	R522, E123, R124
WLRDFL	−12.6	14	2.1, 2.4, 2.5, 2.7, 2.9, 3.0, 3.1, 3.3	E123, Y523, R402, Y360, A356, E384, A354, D377, E162, Q281, K511, H513
FLSQPFF	−11.1	6	2.6, 2.7, 2.8, 3.4, 3.5	Y360, E403, H383, A356, H353, H513
FAWWAGNARL	−10.1	4	2.6, 2.7, 3.3, 3.4	E123, G404, S219
GIHVWHM	−11.5	5	2.5, 2.7, 3.3, 3.4, 3.5	Y360, R124, S517
FIPFLHM	−9.6	1	3.0	E143
VGWLGHPIF	−10.4	7	2.5, 2.7, 3.0, 3.1, 3.2, 3.3	R124, A354, N66, S355
AAIWHW	−10.1	5	2.7, 3.3, 3.4, 3.5	R124, N66, Y62, Y523
MHPFHMLG	−12.7	6	1.7, 2.2, 2.5, 3.2, 3.4	Y360, A356, A354, H353, H383, Q281
LGFHSFGL	−14.2	8	2.5, 2.7, 2.8, 2.9, 3.1, 3.4	Y360, K118, A356, A354, H353, Y523
FTHHMWIGGF	−11.1	6	2.1, 2.6, 3.1, 3.2, 3.4, 3.5	K118, K117, D121, E123, Y360
WTLNPFHMMG	−11.5	8	2.4, 2.5, 2.7, 2.8, 3.2, 3.3, 3.5	R124, R522, Y523, H353, H383, Y394
AAIWHWVY	−6.8	5	2.1, 2.6, 3.0, 3.4, 3.5	R124, N66, Y360, Y394

## Data Availability

The original contributions presented in this study are included in the article. Further inquiries can be directed to the corresponding author.

## Abbreviations

ACE	Angiotensin-converting enzyme
ACEI	Angiotensin-converting enzyme inhibitor
ARB	Angiotensin II receptor blocker
PBS	Phosphate-buffered saline
SEM	Scanning electron microscopy
FTIR	Fourier-transform infrared spectroscopy
LC-MS/MS	Liquid chromatography-tandem mass spectrometry
DPPH	2,2-diphenyl-1-picrylhydrazyl radical
ABTS	2,2′-azino-bis(3-ethylbenzothiazoline-6-sulfonic acid)
AvP	*Apocynum venetum* protein
Av-ACEIP	*Apocynum venetum*-derived ACE inhibitory peptides
FHVAWQGNF	Peptide segment screened from Apocynum venetum hydrolysate
WLRDFL	Lead ACE-inhibitory peptide screened via molecular docking
GAFAAHGAF, SGGIVVHM, VFHNIPM, GGIIVNIREF, WILRDFL, FLSOPEE, FAWWAGNARL, GIHPVWHM, VGIPFLHM, AAINVIHW, MIHPGHPIE, FTIHHWSEG, WILNPFWGG, AAIWHMWVY	Other candidate peptide sequences obtained by virtual screening
